# Effect of fasting plasma glucose level in severe fever and thrombocytopenia syndrome patients without diabetes

**DOI:** 10.1371/journal.pntd.0012125

**Published:** 2024-04-16

**Authors:** Jun Pan, Zhihao Yang, Wen Xu, Shan Tian, Xiaobo Liu, Chunxia Guo

**Affiliations:** 1 Department of Gastroenterology, Taihe Hospital, Hubei University of Medicine, Shiyan, Hubei, China; 2 Department of Infectious Disease, Union Hospital, Tongji Medical College, Huazhong University of Science and Technology, Wuhan, China; University of São Paulo, BRAZIL

## Abstract

Hyperglycemia is correlated with worse in-hospital outcomes in acute infectious diseases such as coronavirus disease 2019 (COVID-19) and severe fever with thrombocytopenia syndrome (SFTS). This study assessed the relationship between fasting plasma glucose (FPG) levels and in-hospital mortality, disease type, and secondary infections among individuals with SFTS without preexisting diabetes. The clinical data and laboratory results upon admission of 560 patients with SFTS without preexisting diabetes meeting the inclusion criteria at Wuhan Union Hospital were collected. FPG levels in surviving patients with SFTS subjects were significantly lower than those in patients with SFTS who had died (P<0.0001). In multivariate Cox regression, high FPG level (≥11.1 mmol/L) was a risk factor independently associated with the in-hospital death of patients with SFTS without preexisting diabetes. Similarly, the FPG levels in general patients with SFTS were significantly lower than those in patients with severe SFTS (P<0.0001). Multivariate logistic regression identified high FPG level (7.0–11.1 mmol/L) as a risk factor independently associated with SFTS severity. While FPG levels were comparable between patients with SFTS with and without secondary infection (P = 0.5521), logistic regression analysis revealed that high FPG levels were not a risk factor for secondary infection in patients with SFTS without preexisting diabetes. High FPG level on admission was an independent predictor of in-hospital death and severe disease in individuals with SFTS without preexisting diabetes. FPG screening upon admission and glycemic control are effective methods for improving the prognosis of patients with SFTS.

## Author summary

Diabetes is a risk factor for a less favorable prognosis in patients with cancer or autoimmune diseases, but few studies have explored the association between fasting plasma glucose (FPG) levels and in-hospital mortality, disease type, and secondary infections among individuals with severe fever and thrombocytopenia syndrome (SFTS). Our study reveal that high FPG level on admission was an independent predictor of in-hospital death and severe disease in individuals with SFTS without preexisting diabetes. FPG screening upon admission and glycemic control are effective methods to assess the prognosis and disease type of patients with SFTS regardless of diabetes status, as most individuals with SFTS are prone to glucose metabolism disorders.

## Introduction

An emerging infectious virus, *Dabie bunyavirus* causes severe fever and thrombocytopenia syndrome (SFTS) [1]. SFTS usually presents with clinical manifestations including liver injury, acute myocardial damage, fever, encephalopathy, and bleeding tendency due to low platelet count. Clinicians have reported SFTS cases worldwide, with mortality rates of 12%–50% [2]. In 2017, the World Health Organization listed SFTS as an emerging infectious disease [3]. Hence, early risk stratification with reliable serum indices is clinically significant for identifying individuals with SFTS with a high risk of in-hospital death.

Survival outcomes of individuals with SFTS largely depend on pre-infection comorbidities [4]. Among the common comorbidities, diabetes is not only a risk factor for susceptibility to SFTS but is also correlated with higher mortality due to immune system impairment [5,6]. Hence, plasma glucose control is critical in the management of patients with SFTS and may improve their prognosis. The presence of diabetes worsens the survival outcome of patients with acute infectious diseases such as Middle -East Respiratory Syndrome [7], coronavirus disease 2019 (COVID-19), and Severe Acute Respiratory Syndrome (SARS)[8]. Acute hyperglycemia is often observed in individuals with SARS-2 with no history of diabetes or glucocorticoid use [9]. This phenomenon most likely occurs due to stress-induced hyperglycemia during an acute inflammatory response [10]. Few clinical studies have reported the influence of hyperglycemia on survival outcomes at admission in SFTS. Only two retrospective studies [11,12] have demonstrated the association of hyperglycemia with increased 28-day mortality; however, both partially included individuals with SFTS and preexisting diabetes. Moreover, whether SFTS virus (SFTSV) influences glucose metabolism in individuals without preexisting diabetes, which further causes secondary infection or even exacerbates the condition, remains unclear.

This retrospective study included only patients with SFTS without preexisting diabetes treated at Wuhan Union Hospital. The patients were divided into three categories based on fasting plasma glucose (FPG) level (<7.0, 7.0–11.1, and FPG≥11.1 mmol/L). The primary aim was to investigate whether FPG levels influenced the clinical outcomes of patients with SFTS without preexisting diabetes. The secondary goal was to determine the relationship between FPG levels, disease severity, and secondary infections in these patients.

## Methods

### Ethics statement

The research protocol related to SFTS was reviewed and endorsed by the Clinical Ethics Committee of Tongji Medical College, Huazhong University of Science and Technology (No. 2023-S093), and this clinical research adhered to the principles of the Declaration of Helsinki. The requirement for written informed consent was waived due to retrospective anonymized data collection.

### Study design

This SFTS cohort included 618 cases treated at Wuhan Union Hospital from March 2017 to November 2022. The Wuhan Union Hospital is the largest medical center for treating SFTS in Central China. SFTSV infection was laboratory-confirmed based on clinical guidelines from the National Health Commission of China [13]. The inclusion criteria were patients with SFTS: (1) confirmed via reverse transcription-polymerase chain reaction (RT-PCR), (2) with FPG data on admission, (3) aged >18 years, and (4) without preexisting diabetes or current trauma. The exclusion criteria were patients with SFTSV (1) co-infected with other viruses such as SARS-1 and SARS-2; (2) lacking FPG data within 24 h of admission due to random blood glucose tests; (3) who received glucocorticoid therapy before transfer to our hospital; (4) patients with a confirmed diagnosis of diabetes or use of anti-diabetic agents.

### Data collection

We collected data on epidemiological features, including geographical area, demographic information, clinical indexes, and laboratory data, from the medical records of patients with SFTS. Two experienced clinicians from our medical center independently checked the clinical data of patients with SFTS, including age, smoking history, alcohol history, sex, chronic medical history (diabetes, chronic kidney disease, cardiovascular disease, and chronic liver disease), clinical symptoms (fever, bleeding tendency, gastrointestinal symptoms, muscle soreness, and muscle tremor), vital signs, and laboratory data (liver and renal function, complete blood count, FPG, blood coagulation, C-reactive protein [CRP], triglyceride [TG], total cholesterol [TC] and procalcitonin [PCT]). Most of the clinical indices were divided into categorical variables based on the reference ranges from Wuhan Union Hospital. Some clinical variables, such as FPG and platelet count (PLT), were divided into categorical variables based on their clinical values. The survival outcomes of most patients with SFTS were recorded at discharge. For patients who were unexpectedly discharged due to clinical deterioration, we performed follow-ups via telephone to confirm their final outcome and date of death.

### Case definitions

FPG levels were measured within 24 h of admission and blood samples were obtained after an overnight fast of ≥8 h. An automatic biochemical analyzer (Beckman Coulter AU5800 Analyzer, USA) was used to measure FPG levels based on the hexokinase method (GLUCOSE kit, RRID:SCR_004098). The main endpoint of this study was in-hospital death. Time of death was calculated from the onset of SFTS symptoms to in-hospital death. Secondary infection was defined as the presence of bacteria or fungi, as determined by sputum or blood culture [14]. SFTS was divided into general and severe types.

### Statistical analysis

The patients were divided into three groups based on FPG levels (<7.0, 7.0–11.1, and FPG≥11.1 mmol/L) at admission [15]. Descriptive statistics were calculated for all clinical data, with categorical variables presented as numbers and percentages and continuous variables presented as means±standard deviations or medians with interquartile ranges (IQRs). The differences in categorical and continuous clinical variable data among the three SFTS groups were compared by chi-square test and one-way analysis of variance (ANOVA), respectively. We analyzed in-hospital mortality using univariate Cox regression analysis to identify potential risk indices. Risk indices with P<0.05 were included in the multivariate Cox regression analysis. Similarly, we performed univariate and multivariate logistic regression analyses to determine whether FPG level was an independent risk index influencing SFTS severity or the occurrence of secondary infections. All statistical calculations were performed using IBM SPSS Statistics for Windows, version 20.0, with P<0.05 considered statistically significant.

## Results

### Baseline features of patients with SFTS without preexisting diabetes

**Fig 1:**
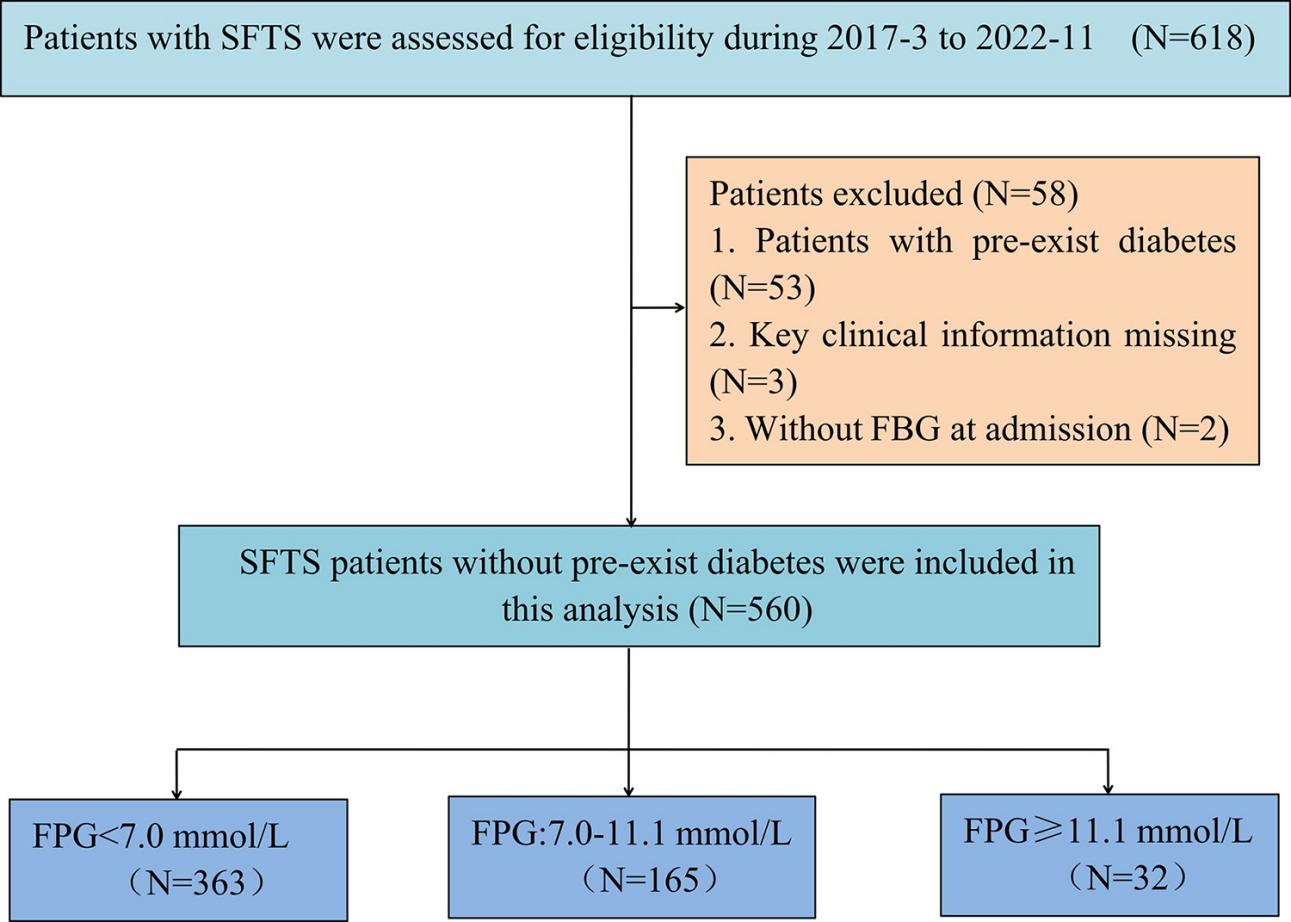
Study flowchart of patients with severe fever with thrombocytopenia syndrome (SFTS) without preexisting diabetes.

A total of 618 patients with SFTS were assessed for study eligibility between March 2017 and November 2022. Among these, 560 patients with SFTS without preexisting diabetes met the inclusion criteria (Fig 1). Their mean age was 61.55±0.45 years and 244 (43.57%) were men. During hospitalization, 73 patients died after treatment in the hospital, while 487 patients eventually recovered, corresponding to a mortality rate of 13.035%. Among the 560 cases of SFTS without preexisting diabetes, 197 (35.18%) showed hyperglycemia and 363 patients (64.82%) had normal FPG levels.

### Comparisons according to FPG levels

**Fig 2:**
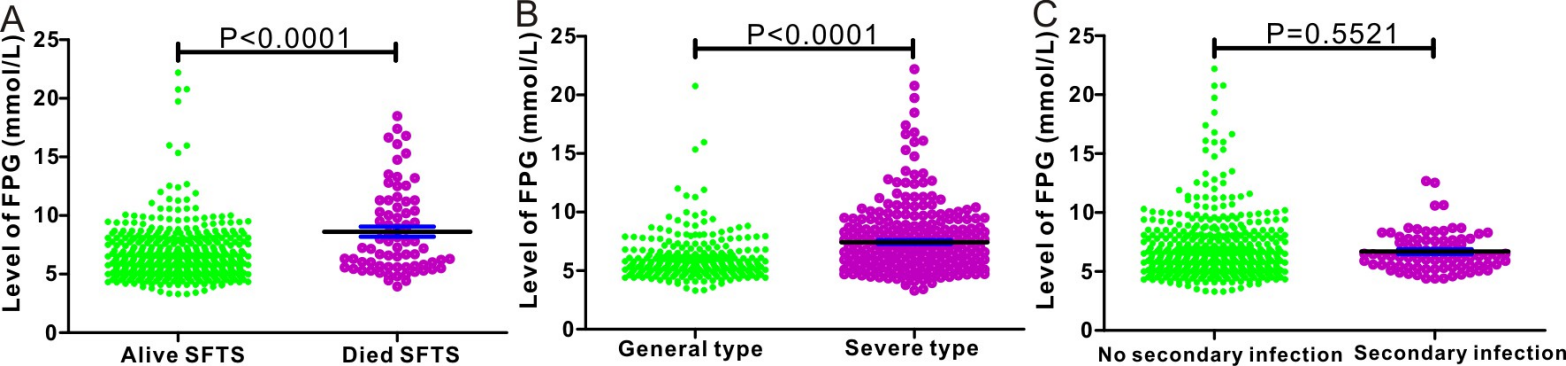
Comparison of fasting plasma glucose (FPG) levels stratified by in-hospital outcomes (**A**), disease severity (**B**), and secondary infection (**C**).

We further divided the 560 cases of SFTS without preexisting diabetes into three groups based on FPG level: group 1 (FPG <7.0 mmol/L), group 2 (FPG 7.0–11.1 mmol/L), and group 3 (FPG ≥11.1 mmol/L). The FPG levels differed significantly different between patients aged <60 and >60 years (P<0.0001). Regarding clinical symptoms, FPG levels differed significantly in the subgroups stratified by bleeding tendency (P<0.0001), gastrointestinal symptoms (P = 0.03), and muscle tremors (P = 0.008). FPG levels also differed significantly different according to CRP (P = 0.005), PCT (P<0.0001), and LDH (P<0.0001) levels. Regarding the endpoints of this clinical study, we utilized scatter plots to compare the FPG levels in patients with SFTS according to living statuses, disease severity, and secondary infections (Table 1). The FPG levels at admission were significantly lower in surviving patients with SFTS than those who died (6.583±0.103 mmol/L vs. 8.623±0.4215 mmol/L, P<0.0001) (Fig 2A). Similarly, the FPG levels were significantly lower in patients with SFTS with general type than those with severe type (6.094±0.128 mmol/L vs. 7.435±0.1583 mmol/L, P<0.0001, Fig 2B). FPG levels were comparable between patients with SFTS with and without secondary infection (6.689±0.1923 mmol/L vs. 6.876±0.1227 mmol/L, P = 0.5521) (Fig 2C).

**Table 1:** Baseline features of SFTS individuals without pre-existed diabetes.

Clinical feature		FPG (<7.0mmol/L)	FPG (7.0–11.1mmol/L)	FPG (> = 11.1mmol/L)	X^2^ value	P value
Gender	Female	200	97	19		
	Male	163	68	13	0.749	0.688
Age	<60	159	161	14		
	≥60	204	104	18	18.245	<0.0001
Bleeding tendency	No	207	75	12		
	Yes	49	41	13	20.337	<0.0001
Digestive symptoms	Yes	65	16	8		
	No	285	139	24	7.034	0.03
Respiratory system performance	No	170	66	15		
	Yes	103	47	6	1.397	0.497
Muscle soreness	No	173	67	15		
	Yes	102	64	10	5.09	0.078
muscle tremor	No	208	81	14		
	Yes	37	23	10	9.692	0.008
lymphadenopathy	No	200	82	15		
	Yes	57	32	6	1.72	0.423
Living status	Alive	330	143	14		
	Dead	33	22	18	38.904	<0.0001
Disease severity	General	194	44	7		
	Severe	169	121	25	39.65	<0.0001
Secondary infection	No	315	137	30		
	Yes	48	28	2	3.278	0.194
CRP	<4 mg/L	172	50	8		
	≥4 mg/L	128	71	13	10.573	0.005
PCT	<0.5 ng/ml	253	84	14		
	≥0.5 ng/ml	98	72	15	19.903	<0.0001
WBC	3.5–5.5G/L	111	53	13		
	Abnormal range	252	112	19	1.402	0.496
PLT	< 30G/L	202	46	12		
	30–50 G/L	61	41	11		
	>50 G/L	100	78	18	40.488	<0.0001
ALT	≤ 40U/L	78	18	6		
	> 40U/L	285	147	26	8.528	0.014
AST	≤ 60U/L	27	12	1		
	> 60U/L	326	153	31	1.097	0.641
ALB	< 30 g/L	102	25	4		
	≥30 g/L	261	140	28	12.859	0.02
LDH	≤500 U/L	118	22	1		
	>500 U/L	242	140	29	29.878	<0.0001
TG	< 1.7 mmol/L	82	26	4		
	≥1.7 mmol/L	159	64	18	2.786	0.248
TC	< 5.2mmol/L	235	83	22		
	≥5.2mmol/L	6	7	0	6.993	0.051
CK	< 140 U/L	66	19	1		
	≥140 U/L	294	143	29	8.725	0.013
CTnI	< 26.2 ng/L	64	22	1		
	≥26.2 ng/L	220	129	29	9.098	0.011
CK-MB	< 6.6 ng/mL	204	98	13		
	≥6.6 ng/mL	59	34	14	11.213	0.004
ferritin	≤2000 ug/L	48	11	1		
	>2000 ug/L	188	89	16	6.523	0.038
APTT	< 43.5 S	98	33	3		
	≥43.5 S	258	131	29	7.453	0.024
PT	< 14.2S	312	136	22		
	≥ 12S	43	28	10	8.296	0.016

### Association between FPG levels on admission and in-hospital death

**Fig 3:**
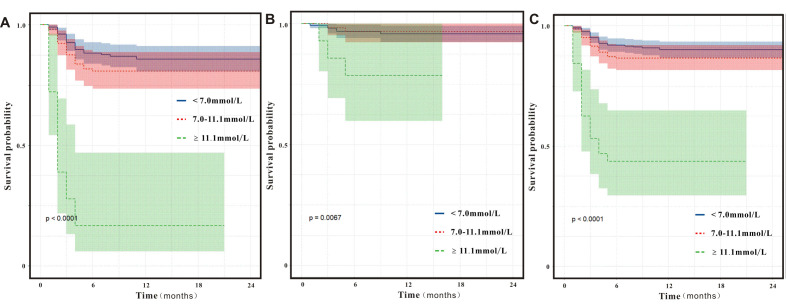
Impact of fasting plasma glucose (FPG) levels at admission on in-hospital death in patients with severe fever with thrombocytopenia syndrome (SFTS) without preexisting diabetes. Kaplan–Meier survival curves of patients with SFTS (**A**) according to FPG levels at admission, (B) aged <60 years, and (**C**) aged ≥60 years.

**Fig 4:**
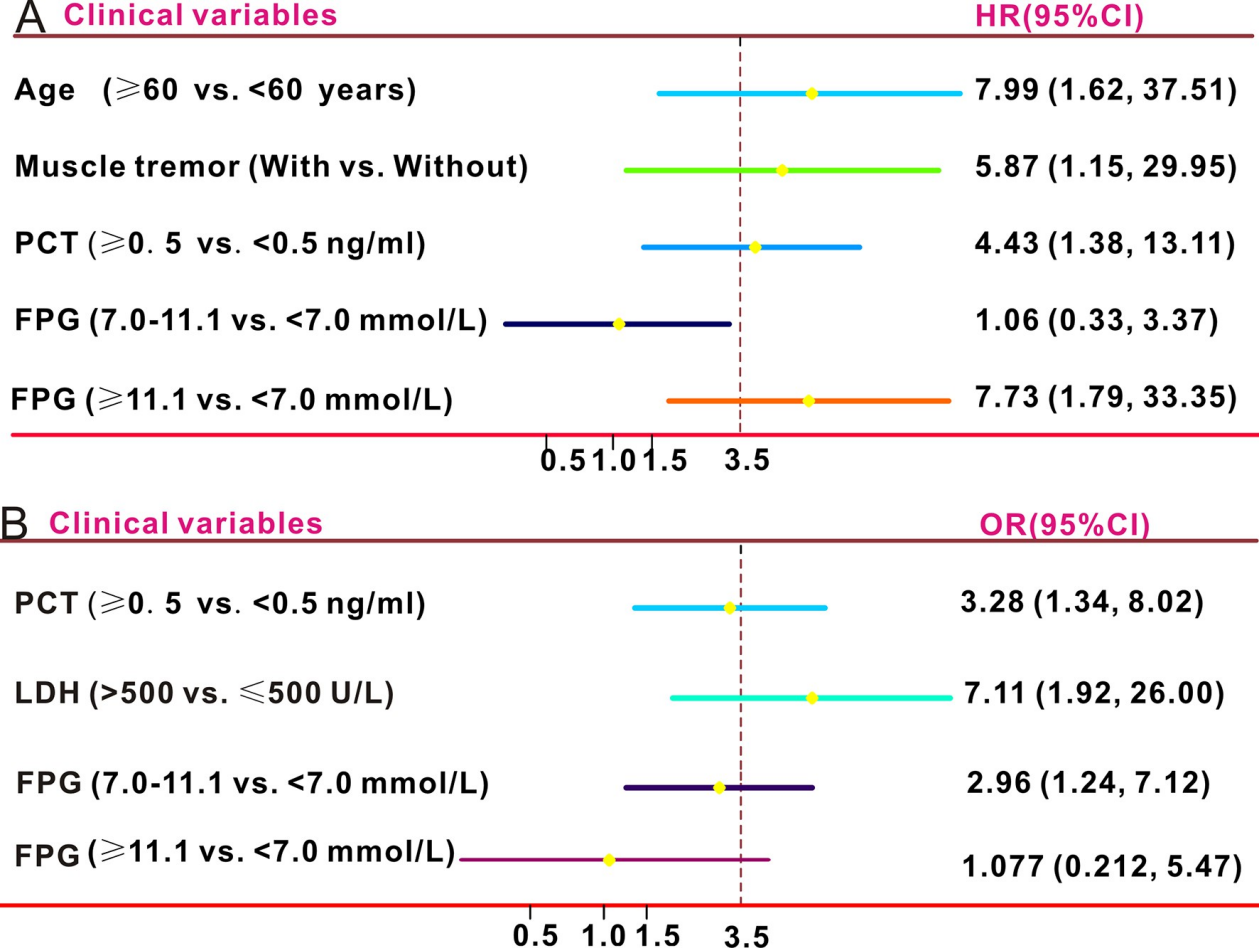
Forest plots of the influence of fasting plasma glucose (FPG) levels on in-hospital mortality and disease severity among patients with severe fever with thrombocytopenia syndrome (SFTS) without preexisting diabetes. **A**. Independent risk factors for in-hospital death by multivariate Cox regression analysis. **B**. Independent risk factors for disease severity by multivariate logistic regression analysis.

Fig 3A shows the Kaplan-Meier curve of patients with SFTS stratified by FPG levels at admission. Compared to patients with FPG <7.0 mmol/L, those with FPG levels of 7.0–11.1 mmol/L exhibited a higher in-hospital death rate; however, the difference was not statistically significant after log-rank tests (hazard ratio [HR] = 1.471, 95% confidence interval [CI]: 0.857–2.523, P = 0.161). Similarly, the in-hospital death rate was higher among patients with FPG levels ≥11.1 mmol/L compared to that in patients with FPG levels <7.0 mmol/L (HR = 8.778, 4.931–15.625, P<0.0001). Univariate Cox regression revealed that age (≥60 years, HR = 4.309, 95%CI: 2.269–8.182, P<0.0001), bleeding tendency (HR = 2.385, 95%CI: 1.38–4.123, P = 0.002), respiratory symptom (HR = 1.833, 95%CI: 1.028–3.27, P = 0.04), PCT (HR = 4.621, 95%CI: 2.761–7.734, P = 0.012) and FPG (HR = 8.778, 95% CI: 4.931–15.625, P<0.0001) were risk factors for in-hospital death among patients with SFTS without preexisting diabetes (Table 2). After adjusting for some clinical variables, age (≥60 years), tremor, and high PCT and FPG levels (≥11.1 mmol/L) were independently associated with in-hospital death among these patients (Fig 4A). As age was also an independent risk factor for in-hospital death in these patients, we conducted a subgroup analysis based on age. Higher FPG levels on admission were associated with an increased in-hospital mortality rate in patients aged <60 years (P = 0.0067) and ≥60 years (P<0.0001, Fig 3B and 3C). In summary, high FPG levels on admission were a potent risk factor for in-hospital mortality among individuals with SFTS without preexisting diabetes.

**Table 2:** Univariate and multivariate Cox regression analysis of prognosis of SFTS individuals without pre-existed diabetes.

Clinical variable		HR	95%CI	P value	HR	95%CI	P value
Gender	0.962		0.605–1.531	0.872			
Age	4.309		2.269–8.182	<0.0001	7.799	1.622–37.505	0.01
Bleeding tendency	2.385		1.38–4.123	0.002	0.086	0.007–1.085	0.058
Digestive symptom	3.946		0.965–16.145	0.056			
Respiratory symptom	1.833		1.028–3.27	0.04	0.937	0.257–3.42	0.921
soreness	1.882		1.1–3.22	0.021	1.081	0.273–4.284	0.912
tremor	4.265		2.433–7.478	<0.0001	5.865	1.149–29.946	0.033
lymphadenopathy	1.29		0.677–2.457	0.439			
Secondary infection	6.336		1.553–25.841	0.01	0.144	0.018–1.158	0.069
CRP	5.084		2.466–10.478	<0.0001	2.8	0.922–8.504	0.069
PCT	4.621		2.761–7.734	<0.0001	4.248	1.377–13.106	0.012
WBC	1.519		0.883–2.614	0.131			
PLT	1.964		1.092–3.533	0.024	2.158	0.578–8.059	0.252
	3.4		1.89–6.117	<0.0001	0.681	0.177–2.626	0.577
ALT	1.875		0.899–3.907	0.094			
AST	2.28		0.718–7.24	0.162			
ALB	1.305		0.729–2.337	0.371			
LDH	3.905		1.693–9.009	0.001	1.821	0.205–16.161	0.591
TG	1.691		0.932–3.067	0.084			
TC	1.424		0.348–5.829	0.623			
CK	4.452		1.401–14.146	0.011	1.061	0.123–9.185	0.957
CTnI	6.883		1.685–28.124	0.007	3.993	0.412–38.689	0.232
CKMB	2.768		1.672–4.582	<0.0001	0.801	0.268–2.397	0.692
ferritin	26.981		0.785–929.271	0.068			
FPG	1.471		0.857–2.523	0.161	1.064	0.327–3.467	0.918
	8.778		4.931–15.625	<0.0001	7.732	1.793–33.346	0.006
APTT	7.681		2.417–24.408	0.001	5.132	0.479–54.934	0.176
PT	2.965		1.806–4.867	<0.0001	2.52	0.836–7.596	0.101

### Relationship between FPG and disease severity

The secondary study outcome was SFTS severity. As mentioned above, patients with severe SFTS exhibited higher FPG levels on admission than those with general SFTS. Hence, we performed univariate and multiple logistic regression analyses to determine whether the FPG level on admission was an independent risk factor affecting SFTS severity in patients without preexisting diabetes. Univariate logistic regression identified risk factors for severe SFTS, including age and PCT, LDH, and FPG levels at admission (Table 3). After adjusting for potential factors, multivariate logistic analysis (Fig 4B) revealed that high FPG levels (7.0–11.1 mmol/L) at admission was an independent risk factor of the severe type of SFTS patients (odds ratio [OR] = 2.964, 95%CI 1.235–7.116, P = 0.015). The possible reason might be that high FPG is usually the consequence of an infection due to the release of counter-regulators.

**Table 3:** Univariate and multivariate logistic regression analysis of disease type of SFTS individuals without pre-existed diabetes.

Clinical variable	OR	95%CI	P value	OR	95%CI	P value
Gender	1.053	0.752–1.475	0.764			
Age	2.029	1.1442–2.857	<0.0001	1.361	0.613–3.024	0.449
Bleeding	3.095	1.857–5.16	<0.0001	1.923	0.538–6.872	0.314
Digestive symptom	1.676	0.962–2.921				
Respiratory symptom	1.737	1.151–2.62	0.009	0.547	0.203–1.47	0.232
soreness	1.888	1.27–2.808	0.002	1.666	0.59–4.699	0.335
tremor	2.977	1.649–5.375	<0.0001	1.421	0.346–5.83	0.626
lymphadenopathy	0.876	0.551–1.391	0.574			
Secondary infection	1.665	1.006–2.756	0.047	0.724	0.217–2.419	0.6
CRP	1.764	1.208–2.577	0.003	0.464	0.211–1.021	0.056
PCT	4.086	2.731–6.113	<0.0001	3.277	1.339–8.017	0.009
WBC	0.953	0.665–1.365	0.792			
ALT	1.462	1.214–1.762	<0.0001	0.396	0.577–4.023	1.523
AST	1.406	1.179–1.676	<0.0001	0.193	0.079–1.668	0.363
ALB	1.616	1.33–1.963	<0.0001	0.053	0.143–1.012	0.381
LDH	1.777	1.453–2.174	<0.0001	0.003	1.915–26.37	7.107
TG	0.953	0.605–1.499	0.834			
TC	0.590	0.156–2.235	0.437			
CK	1.533	1.273–1.845	<0.0001	0.248	0.184–1.548	0.534
CTnI	1.643	1.335–2.023	<0.0001	0.96	0.345–2.747	0.974
CKMB	2.344	1.549–3.545	<0.0001	0.305	0.634–4.294	1.65
ferritin	3.363	1.081–1.718	0.009	0.297	0.136–1.84	0.5
FPG	2.75	1.948–3.883	<0.0001	0.015	1.235–7.116	2.964
	3.571	1.545–8.257	0.003	0.929	0.212–5.47	1.077
APTT	1.679	1.378–2.048	<0.0001	0.253	0.657–4.934	1.801
PT	3.5	2.073–5.91	<0.0001	0.688	0.417–3.765	1.253

### Correlations between FPG levels and secondary infections

As secondary infection is a common complication in patients with diabetes, we utilized logistic regression to investigate the potential correlation of high FPG levels at admission with secondary infection among individuals with SFTS without preexisting diabetes. Logistic regression analysis did not identify high FPG level as a risk factor for the occurrence of secondary infection among these patients (OR_1_ = 2.286, 95%CI:0.529–9.874, P = 0.268; OR_2_ = 3.066, 95%CI:0.692–13.575, P = 0.14) (Table 4). One possible explanation for this finding might be the routine use of antibiotics for prevention in our medical center in individuals with severe SFTS.

**Table 4:** Univariate logistic regression analysis of predictors for secondary infection among SFTS individuals without pre-exist diabetes.

Clinical variables	OR	95%CI	P value
Gender	1.001	0.618–1.621	0.997
Age	1.999	1.182–3.378	0.01
Bleeding	3.155	1.652–6.025	0.001
Digestive symptom	1.608	0.618–4.188	0.331
Respiratory symptom	1.892	1.032–3.467	0.039
soreness	2.354	1.316–4.209	0.004
tremor	2.906	1.416–5.964	0.004
lymphadenopathy	1.728	0.866–3.451	0.121
severity	1.665	1.006–2.756	0.407
CRP	1.798	0.991–3.265	0.054
PCT	1.52	0.93–2.484	0.095
WBC	0.749	0.455–1.232	0.255
PLT	2.965	1.646–5.341	<0.0001
	1.325	0.735–2.39	0.349
ALT	1.263	0.655–2.434	0.486
AST	1.503	0.578–3.913	0.403
ALB	1.331	0.73–2.427	0.351
LDH	1.749	0.93–3.288	0.083
TG	0.679	0.368–1.254	0.216
TC	0.735	0.09–6.004	0.774
CK	1.928	0.854–4.354	0.114
CTnI	4.654	1.42–15.25	0.011
CKMB	0.758	0.385–1.494	0.423
ferritin	3.004	0.898–10.053	0.074
FPG	2.286	0.529–9.874	0.268
	3.066	0.692–13.575	0.14
APTT	1.872	0.978–3.583	0.058
PT	0.979	0.492–1.947	0.952

## Discussion

Diabetes is a risk factor for a less favorable prognosis in patients with cancer or autoimmune diseases. Recent evidence has also suggested that preexisting diabetes increases the risk of worse in-hospital survival outcomes among individuals with acute infectious diseases such as COVID-19 and SFTS. However, the effects of admission FPG levels on SFTS progression in patients without preexisting diabetes are unknown. Our analysis is the first clinical report from a large SFTS cohort to investigate the association between FPG levels on admission, prognosis, and SFTS severity among patients without preexisting diabetes. The 560 patients with SFTS and no preexisting diabetes showed an increased prevalence of high FPG levels at admission. A high FPG level was significantly correlated with severe disease and worse in-hospital outcomes among patients with SFTS without preexisting diabetes. Our results suggest that measuring FPG level on admission in patients with SFTS would allow better risk stratification due to the close relationship between FPG level and prognosis and guide the intensive treatment of patients with SFTS at high risk. High FPG is an indirect marker of the severity of the SFTS infection, and that the SFTS infection per severity is responsible for the worse outcome of SFTS individuals.

FPG levels are reportedly correlated with in-hospital death in patients with acute infectious disease without preexisting diabetes. In their multivariate analysis, Andrea et al [16] reported that patients with COVID-19 and high FPG levels without preexisting diabetes were 2.06-fold times more likely to die than those with low FPG levels (HR = 2.06, 95%CI 1.45–2.92, P<0.001). The results of their clinical study indicated that a high FPG level was an independent risk biomarker for in-hospital mortality in patients with COVID-19 without preexisting diabetes. Moreover, Wang et al [17] reported that elevated FPG levels (≥7.0 mmol/L) on admission reliably predicted in-hospital mortality in patients with COVID-19 without preexisting diabetes. Like COVID-19, SFTS is an acute inflammatory response caused by viral infections and metabolic disorders. In their cohort study of 77 cases of SFTS, Zhang et al.[12] concluded that the detection of blood glucose levels can help in assessing the hypercoagulability and inflammatory status of these patients subjects. A multicenter SFTS cohort study [11] indicated that acute hyperglycemia after admission was a strong predictor of in-hospital death in female patients with SFTS. However, these two clinical studies on SFTS and blood glucose levels included individuals with preexisting diabetes and newly diagnosed diabetes. To our knowledge, the present cohort study is the first to reveal the potential correlation between FPG levels on admission and the in-hospital prognosis of patients with SFTS without preexisting diabetes. Multivariate Cox regression analysis indicated that elevated FPG concentrations were significantly correlated with an increased risk of death among patients with SFTS without preexisting diabetes. Based on our findings, we propose that FPG testing and plasma glucose control are important in patients with SFTS, even in those without preexisting diabetes, and may help improve their in-hospital prognosis.

SFTS is an acute inflammatory disease caused by SFTSV, and acute hyperglycemia upon admission is common. In our cohort, 35.18% of patients with SFTS without preexisting diabetes exhibited acute hyperglycemia, while 64.82% showed normal plasma glucose levels, which are more likely to be ascribed to stress hyperglycemia. Cytokine storms contribute to SFTS progression and deterioration [18] and cause multiple organ failure. Cytokine storms can induce stress hyperglycemia in COVID-19[19]. Stress hyperglycemia in acute illness is a hallmark of insulin insufficiency, which is associated with increased levels of free fatty acids and lipolysis, and a high risk of ketoacidosis. Acute hyperglycemia may also be associated with insulin resistance. Acute systemic inflammation caused by cytokine storms can induce or aggregate insulin resistance [20]. The hyperglycemia in patients with acute illness appears to be related to a variety of stress mechanisms [21]. Stress hyperglycemia is a transient increase in blood glucose during acute stress or illness. The release of counterregulatory hormones, such as catecholamine, is involved in insulin resistance with deceased glycogen break down and increased hepatic glucose output. The continuous hyperglycemia which seems to regulate the acute stress situation, is harmful to the body because it induces oxidative stress-related injury, mitochondrial impairment and dysfunction of several cellular channels, which might explain that patients in acute stress with hyperglycemia tend to exhibit worse survival outcome [22]. Hence, blood glucose control is vital for managing patients with SFTS, especially those without preexisting diabetes, as blood glucose control is easily ignored.

Ours is the first clinical analysis to reveal the relationship between FPG levels and secondary infections in patients with SFTS. Increased FPG levels are a metabolic response to acute stress in most critically ill patients. High FPG levels are also significantly associated with increased risks of secondary infections [23,24]. High blood glucose levels inhibit neutrophil function, impair macrophage phagocytosis, and decrease immunoglobulin-mediated bacterial opsonization [25] and can negatively regulate major components of immune response and cause aberrant glycosylation of enzymes and immunoglobulins that play critical roles in adaptive immunity [26]. However, we did not identify FPG level as a risk factor for secondary infection among 560 patients with SFTS. One likely explanation for this non-significant association is the routine clinician of antibiotics to these patients to prevent sepsis upon admission from our medical center.

Diabetes and glucocorticoid therapy are the most important factors affecting FPG levels. We excluded patients with SFTS with preexisting diabetes or who had received glucocorticoid therapy before transfer to our hospital. Hence, our finding of the correlation between FPG levels and the prognosis of individuals was robust. However, our analysis had two limitations. First, HbA1c levels are not routinely measured upon admission in individuals with SFTS. HbA1c reflects the average blood glucose level over the past three months; thus, we could not determine the average blood glucose level of these individuals before infection with SFTSV. Second, Wuhan is not an area endemic for SFTSV, and all individuals were from mountainous areas. Most patients with general-type SFTS receive treatment at local hospitals. Hence, most cases in our hospital were severe, leading to a selection bias. Therefore, prospective clinical studies of SFTS in multiple hospitals are needed.

## Conclusion

A higher FPG level on admission was an independent predictor of in-hospital death and severe disease type in individuals with SFTS without preexisting diabetes. FPG screening upon admission and glycemic control are effective methods to assess the prognosis and disease type of patients with SFTS regardless of diabetes status, as most individuals with SFTS are prone to glucose metabolism disorders.

## Supporting information

S1 Data Raw data of 560 patients with severe fever with thrombocytopenia syndrome. (XLS)
